# Prefrontal Theta-Phase Synchronized Brain Stimulation With Real-Time EEG-Triggered TMS

**DOI:** 10.3389/fnhum.2021.691821

**Published:** 2021-06-21

**Authors:** Pedro Caldana Gordon, Sara Dörre, Paolo Belardinelli, Matti Stenroos, Brigitte Zrenner, Ulf Ziemann, Christoph Zrenner

**Affiliations:** ^1^Department of Neurology and Stroke, University of Tübingen, Tübingen, Germany; ^2^Hertie Institute for Clinical Brain Research, University of Tübingen, Tübingen, Germany; ^3^CIMeC, Center for Mind/Brain Sciences, University of Trento, Rovereto, Italy; ^4^Department of Neuroscience and Biomedical Engineering, Aalto University School of Science, Espoo, Finland

**Keywords:** EEG, TMS, prefrontal cortex, brain-state dependent stimulation, non-invasive brain stimulation, theta rhythm, brain oscillations

## Abstract

**Background:**

Theta-band neuronal oscillations in the prefrontal cortex are associated with several cognitive functions. Oscillatory phase is an important correlate of excitability and phase synchrony mediates information transfer between neuronal populations oscillating at that frequency. The ability to extract and exploit the prefrontal theta rhythm in real time in humans would facilitate insight into neurophysiological mechanisms of cognitive processes involving the prefrontal cortex, and development of brain-state-dependent stimulation for therapeutic applications.

**Objectives:**

We investigate individual source-space beamforming-based estimation of the prefrontal theta oscillation as a method to target specific phases of the ongoing theta oscillations in the human dorsomedial prefrontal cortex (DMPFC) with real-time EEG-triggered transcranial magnetic stimulation (TMS). Different spatial filters for extracting the prefrontal theta oscillation from EEG signals are compared and additional signal quality criteria are assessed to take into account the dynamics of this cortical oscillation.

**Methods:**

Twenty two healthy participants were recruited for anatomical MRI scans and EEG recordings with 18 composing the final analysis. We calculated individual spatial filters based on EEG beamforming in source space. The extracted EEG signal was then used to simulate real-time phase-detection and quantify the accuracy as compared to post-hoc phase estimates. Different spatial filters and triggering parameters were compared. Finally, we validated the feasibility of this approach by actual real-time triggering of TMS pulses at different phases of the prefrontal theta oscillation.

**Results:**

Higher phase-detection accuracy was achieved using individualized source-based spatial filters, as compared to an average or standard Laplacian filter, and also by detecting and avoiding periods of low theta amplitude and periods containing a phase reset. Using optimized parameters, prefrontal theta-phase synchronized TMS of DMPFC was achieved with an accuracy of ±55°.

**Conclusion:**

This study demonstrates the feasibility of triggering TMS pulses during different phases of the ongoing prefrontal theta oscillation in real time. This method is relevant for brain state-dependent stimulation in human studies of cognition. It will also enable new personalized therapeutic repetitive TMS protocols for more effective treatment of neuropsychiatric disorders.

## Introduction

Synchronous oscillatory activity between neuronal populations allows information exchange and the strengthening of connections through neuroplasticity (Harris et al., 2003; Buzsaki and Draguhn, 2004). These neuronal oscillations are ubiquitous in the functioning brain cortex and can be observed with the aid of several tools, including non-invasive recordings such as electroencephalography (EEG) and magnetoencephalography. Different oscillatory patterns predominate in different cortical areas and are modulated by the individual’s states and behavior, suggesting that each oscillatory mode has specific physiological functions. Specifically, oscillatory activity in the theta frequency band (4–7 Hz), which can be found in brain areas such as the prefrontal cortex and hippocampus, has been correlated with several cognitive processes, making it a phenomenon of interest for developing diagnostics and treatment of neuropsychiatric disorders (Lisman and Buzsaki, 2008; Sauseng et al., 2010; Cavanagh and Frank, 2014).

Advances on understanding the relationship between cognition and neuronal oscillations in the theta band have mainly relied on studies in animal models. Early research has demonstrated spatial memory deficits following the loss of theta rhythm in the hippocampus (Winson, 1978). Moreover, the hippocampal theta oscillation has been found to be significantly phase-locked to the neuronal firing of large populations of neurons in the medial prefrontal cortex (Siapas et al., 2005), with different phases corresponding to different states of excitability. Accordingly, neuronal spiking has been observed predominantly in specific theta-phases depending on the brain region (Klausberger et al., 2004; Fujisawa and Buzsaki, 2011), with stimulation applied in different phases of theta oscillation yielding differential profiles of neuroplasticity (Pavlides et al., 1988; Holscher et al., 1997; Hyman et al., 2003). Together, these observations support the notion that different phases of theta oscillations represent distinct excitability states of neuronal populations, which would enable effective neuronal communication and different opportunities of plasticity induction (for comprehensive review see Fries, 2015). Studies in human subjects have confirmed the presence of a marked theta rhythm in the frontal midline EEG channels, originating from the anterior part of the superior frontal gyrus and anterior cingulate cortex (Ishii et al., 1999; Onton et al., 2005). Concomitant EEG measures and task performance confirmed an association between prefrontal theta dynamics and cognition, showing increasing power of theta oscillations and connectivity enhancement within prefrontal cortices, as well as between prefrontal and parietal cortices during tasks that required heavier memory loads (Onton et al., 2005; Sauseng et al., 2007). Intracranial recordings from patients undergoing invasive procedures further support the association between theta rhythm dynamics and cognition in humans, as well as phase-specific preferences for neuronal firing (Kahana et al., 1999; Rizzuto et al., 2006; Rutishauser et al., 2010; Lega et al., 2012; Zavala et al., 2018).

Given the role of different phases of the prefrontal theta oscillation, interfering with this oscillatory mode by applying non-invasive brain stimulation in a phase-specific manner may prove to be a relevant asset for modulating human brain function. This concept has previously been explored by our group, demonstrating that transcranial magnetic stimulation (TMS) in humans evokes differential responses depending on the phase of an ongoing local low-frequency EEG oscillation. Specifically, it has been found that the negative peak of the sensorimotor μ-oscillation represents a state of higher responsivity compared to the positive peak and random phase, as TMS during the negative peak evoked higher-amplitude motor potentials, and repetitive TMS induced long-term potentiation-like effects, which was made possible by using a real-time phase-detection algorithm (Schaworonkow et al., 2018; Zrenner et al., 2018). Here, we aim to develop a method to enable phase-specific stimulation according to the prefrontal theta oscillation.

However, differences in the characteristics of prefrontal theta and sensorimotor μ-oscillations require significant changes to the methods involved in the real-time phase-detection. Firstly, despite theta oscillations being prominently observed in EEG prefrontal regions, the signal-to-noise ratio (SNR) is usually lower than the SNR for the sensorimotor μ-oscillation in the alpha-frequency band. Lower SNR leads to increased vulnerability of the measured signal to interference from other oscillatory sources, either local or via volume conduction, which increases the estimation error of the phase of the theta oscillation of interest, while also decreasing the accuracy of the real-time phase-detection algorithm (Zrenner et al., 2020). Additionally, cortical theta oscillations have been found to occur in limited time lengths, sometimes described as bursts, which can last from a couple of cycles up to 2 seconds, which translates into a signal prone to sporadic shifts in amplitude and phase resetting (Kahana et al., 1999; Rizzuto et al., 2006; Rutishauser et al., 2010). This poses a challenge for any phase-estimation method, as one cannot rely on extrapolations from long windows of data, given that longer windows are more likely to contain periods of interference or a phase reset. Addressing these issues is particularly relevant for designing an accurate real-time phase-detection algorithm aimed at the prefrontal theta oscillation. Therefore, such algorithm should include methods to reliably extract the theta oscillation from the cortical region of interest and to guarantee that the input signal to the real-time system corresponds to a stable oscillation, implying a signal length free from theta amplitude shifts and phase resets, which might compromise proper phase detection.

We propose here adaptations to the real-time phase detection method presented previously (Zrenner et al., 2018), in order to account for the characteristics of the prefrontal theta oscillation, and thereby enable real-time EEG-triggered TMS targeting of specific phases of the theta oscillation. We also assess the benefit of using an individual EEG spatial filter, based on the subject’s anatomical MRI and EEG source activity estimation, designed to extract the signal of interest from the dorsomedial prefrontal cortex (DMPFC) in real time, as opposed to using a standard EEG montage. We also propose additional trigger constraints, which allow the real-time phase detection algorithm to detect instabilities in the theta oscillation, only triggering pulses during stable theta oscillation with adequate amplitude and without phase resets. We hope that our findings will enable future studies into the physiology of the human theta oscillation as well as new theta phase-dependent therapeutic neuromodulation.

## Materials and Methods

### Subjects and Design

Twenty-two healthy individuals were recruited. Inclusion criteria were the absence of past or current psychiatric or neurological diseases. Exclusion criteria were current treatment with drugs acting on the central nervous system, presence or prior history of alcohol or illicit drugs abuse, and pregnancy. Two subjects were excluded due to excessive sleepiness, and two due to excessive eye movements and muscular activity during the experiment. The final sample included 18 subjects (11 female) with a mean age (±1 SD) of 24 ± 3.3 years. All subjects provided written informed consent prior to participation, and the study was conducted in accord with the Declaration of Helsinki approved by the ethics committee of the medical faculty of the University of Tübingen (716/2014BO2).

### EEG and TMS

Scalp EEG was recorded with a 126-channel TMS compatible Ag/AgCl sintered ring electrode cap (EasyCap GmbH, Germany) in the International 10-5 EEG system arrangement (Oostenveld et al., 2011), with reference and ground electrodes placed at positions FCz and CPz, respectively. A 24-bit biosignal amplifier was used for EEG recordings, at a sampling rate of 5 kHz (NeurOne Tesla with Digital Out Option, Bittium Biosignals Ltd., Finland), in DC mode. TMS was delivered using a MagPro XP Stimulator (MagVenture A/S, Denmark) connected to a figure-of-eight coil (Cool-B65, inner coil winding diameter 35 mm) with biphasic pulses of 300 μs width.

### Experimental Session

The experiment was conducted in a quiet room with the subjects sitting comfortably in a reclined chair, instructed to keep their eyes open. Experimental measurements involved a series of 3 EEG recordings. The first recording consisted of 8 min of resting-state EEG. This signal was used for the covariance matrix calculation, required for the design of the individualized source-based spatial filter, as explained in the next sub-section. This recording was also used to later test different parameters of the real-time phase estimation algorithm, in simulating real-time phase estimation offline, also explained below.

The second recording also involved resting-state EEG, but here we used the real-time phase-estimation algorithm to mark the EEG recording in real-time whenever the conditions for triggering at either the negative or positive peak of the ongoing theta oscillation were met. Sufficient data was recorded to include 100 markers for each phase condition. The procedure was designed to enable the quantification of the real-time algorithm’s accuracy by comparing the predicted phase with a post-hoc “gold-standard” phase estimate. This was necessary as the real-time algorithm is essentially “predictive,” relying on preceding signal only, which reduces the accuracy of the phase estimate (Blackwood et al., 2018; Zrenner et al., 2020). By analyzing the same signal *post hoc*, including data before and after the time point of interest, with standard signal-processing methods, the accuracy of the real-time estimate for a given signal can be assessed (details are provided below). It is important that the signal contains only trigger markers, but not actual TMS pulses, as stimulus artifacts and evoked cortical responses distort the post-stimulus signal, which cannot then be used for a *post hoc* phase estimate. Nonetheless, this method enables a reliable estimate of actual phase targeting accuracy, given that the marker placement in non-stimulated epochs follows exactly the same procedure and constraints as for the stimulated epochs (Bergmann et al., 2012; Zrenner et al., 2018).

The third part of the experiment consisted of the application of 480 EEG-triggered single biphasic TMS pulses to the DMPFC using the real-time phase detection algorithm to trigger pulses to either the positive peak, negative peak or random phases of the theta oscillation, 160 pulses per condition. The coil was separated from the scalp using an 11 mm plastic spacer that was mounted on the EEG electrodes to prevent direct contact of the TMS coil with the electrodes and reduce possible artifacts (Ruddy et al., 2017). Pulses were applied with an intensity of 120% of the resting motor threshold (RMT) (Groppa et al., 2012) and a minimum interstimulus interval of 2.5 s.

### Imaging and Head Model

Extraction of brain activity from DMPFC was achieved by using an individual source-based spatial filter (filter W_ind_). For this purpose, all subjects underwent MRI using a 3T Siemens PRISMA scanner, with T1- and T2-weighted anatomical sequences, required for the EEG forward model. The neuronavigation system (Localite GmbH, Sankt Augustin, Germany) was used to locate the left DMPFC (Dunlop et al., 2015), identified by the MNI coordinates (−4, 52, 36) (Baetens et al., 2017; Piva et al., 2019). Individual MRIs were segmented and meshed using the Fieldtrip toolbox (Oostenveld et al., 2011), which relies on the software packages FreeSurfer and HCP workbench (Fischl, 2012). Meshes were imported into MATLAB R2018b (Mathworks Ltd., United States) and a forward model for EEG was built using a customized pipeline (Stenroos and Sarvas, 2012; Stenroos and Nummenmaa, 2016). Positions of all 126 electrodes were pinpointed manually using the neuronavigation system, and then projected onto the scalp surface mesh. A three-compartment volume conductor model was constructed using the boundary element method comprising the intracranial space (conductivity 0.33 S/m), skull (0.0041 S/m), and scalp (0.33 S/m). Cortical source activity was represented as primary current density on the boundary of white and gray matter, discretized into approximately 16,000 cortical source dipoles, each oriented perpendicular to the cortical surface. Signal topographies for all these dipoles were computed yielding a 126 × 16,000 leadfield matrix *L*, which quantifies how the source activity at each cortex location contributes to the voltage distribution on the sensor array. Cortical dipoles within 1 cm diameter centered in the left DMPFC coordinates were set as the region of interest for the EEG source activity estimation.

### EEG Source Activity Estimation

A linear constrained minimum variance (LCMV) beamformer was used to estimate source activity at the relevant locations in source space (Van Veen et al., 1997). With constrained source orientations, the source amplitude *s(r,t)* in location *r* at time-instant *t* is obtained by

(1) $$s\,\left(r,t\right)=w^{T}\,\left(r\right)\,E\,\left(t\right),$$

where *E* is the array of measured EEG signals, and *w* is the spatial filter vector defined as

(2) $$w=\frac{l^{T}\,\left(r\right)\,C^{-1}}{l^{T}\,\left(r\right)\,C^{-1}\,l\,\left(r\right)},$$

where *l(r)* is the topography of a elementary source dipole at location *r*, i.e., the corresponding column of the leadfield matrix *L*, and *C* is the signal covariance matrix, which was in this study calculated on 8 min of resting-state EEG data.

Spatial filters *W* and estimated time-course *s* were calculated for the selected dipoles in the region of interest. The individual filter W_ind_ is constructed using only those columns of the leadfield matrix *L* corresponding to the dipoles located within the left DMPFC. As this procedure depends on the covariance matrix of the acquired signal and on source topographies, which in turn depend on the head conductivity geometry and sensor positions with respect to the sources, each spatial filter is specifically calculated for each single session and subject. For the purpose of plotting, the results obtained from source estimation for each subject was then pooled and warped into a common MNI space for group average across subjects.

We used the resting state EEG data to estimate the source of the theta oscillation in the cortical surface by performing a spectral analysis at source level, using the individual head models and LCMV beamforming. Spectral power was estimated using the multi-taper method on contiguous data segments 5 s long with 5 tapers and a time half-bandwidth parameter of 3 yielding a power spectral density estimate of the full spectrum. Fractal (aperiodic) background noise was estimated using the Irregular Resampling Auto-Spectral Analysis (IRASA) method (Wen and Liu, 2016) with factors 1.1–2.9 in steps of 0.1 and excluding 2.0, as implemented by the Fieldtrip toolbox. SNR was computed by subtracting the fractal (aperiodic) component from the full spectrum (Donoghue et al., 2020).

### Spatial Filters Comparison

In order to test the relevance of using an individual source-based spatial filter, the filter W_ind_, we compared its properties with that from three non-individual spatial filters. The first of these filters involved the grand-average of the coefficient weights of the filter W_ind_, channel by channel, across all subjects, resulting in the filter W_avg_, which was applied as a generic spatial filter for all subjects. A simpler approach was to use the electrode with the highest coefficient weight in the filter W_avg_ as the center of a Hjorth montage (electrode AFF1h; coefficient weight = 1), with surrounding electrodes suppressing the signal (AFp1, AFF2h, FFC1h, AFF5h; coefficient weights = −1/4), resulting in the filter W_H_, similarly to what has been done to detect the sensorimotor μ-oscillation (Zrenner et al., 2018). Finally, the simplest method was to consider solely the signal from the AFF1h electrode, resulting in the filter W_A_. These spatial filters were compared with regard to the expected cortical areas they are sensitive to, performed by multiplying each individual filter by the whole leadfield matrix *L*, yielding a sensitivity profile over the cortical mesh. Furthermore, sensitivity profiles were normalized within subjects by means of a z-transform, subtracting the sensitivity of each dipole by the individual’s average and divided by its standard deviation. The same procedure was applied to the individual electrodes in the sensor level, to better illustrate the conformation of the spatial filters. The correlation coefficient between the filters’ sensitivity profiles across all the cortical surface was calculated on the individual subject level, as an estimate of similarity between these filters. The resulting correlation coefficients were then statistically compared. To summarize, the filters of interest were: (A) W_A_, single electrode (AFF1h), with an average reference, (B) W_H_, Hjorth-style Surface Laplacian montage centered on AFF1h (Hjorth, 1975; Tenke and Kayser, 2012), (C) W_avg_, Non-individual beamforming (the average of the individual filter across all subjects), (D) W_ind_, Individual source based spatial filter,

Each spatial filter was applied to resting-state EEG data in order to characterize and compare the resulting oscillation. The signal was first down-sampled to 250 Hz as done in the real-time phase estimation algorithm (see below). In order to compare the signal resulting from each filter with regards to their spectral distribution, a spectral analysis was performed using the same IRASA procedure as described above, allowing an estimate of the SNR. Total SNR in the theta band (5–8 Hz) from each filter was then statistically compared between the signals. The following analysis was performed to assess the stability of the theta oscillation extracted using different spatial filters, estimating the signal length of the theta oscillation between phase resets: the signal was zero-phase (forward and backward) filtered using a theta band-pass filter (5–8 Hz pass-band, FIR order 250) and Hilbert transformed to yield the analytic signal. The complex angle (corresponding to instantaneous phase) was unwrapped (using the Matlab “unwrap” command) yielding phase progression of the signal with the slope corresponding to instantaneous frequency (radians/Hz), and the second derivative as a measure of the stability of the oscillation’s phase progression: A value around zero represents stable phase progression, whereas phase slips are indicated by brief deviations from zero (possibly representing physiological phase resetting; see also Freeman: Origin, structure, and role of background EEG activity. Part 1 and 2; Freeman,2004a,b, or interference from other sources). To detect phase slips in the signal using this procedure, the absolute value of the second derivative was taken and a threshold of higher than 5 times above the median was defined. From this, the distribution of the durations of stable periods between phase-slips was determined and statistically compared between the spatial filters (after log-transformation to reduce skew of the distribution).

### Real-Time Phase Estimation Algorithm

EEG theta phase was estimated in real-time by downsampling the spatially filtered signal to 250 Hz and analyzing sliding windows of data with length of 256 samples (1,024 ms), applying the following steps every 4 ms to yield an instantaneous phase estimate: (1) zero-phase forward and backward filtering with an FIR 5–8 Hz band-pass filter of order 80, (2) removal of 35 samples from the epoch’s window closest to the marker in order to reduce filtering edge effects, (3) autoregressive forward, Yule-Walker method, prediction of order 15 and the total predicted interval of 268 ms (140 ms for the removed edge, and 128 ms into the future to avoid edge effects from the Hilbert transform), (4) Hilbert transform. TMS was triggered when the estimated phase fell into a predetermined range and two further conditions were met: a minimum of 1 s had passed since the previous stimulus and no signal artifacts were detected (explained below). We chose the theta band as 5–8 Hz instead of the classical 4–7 Hz due to pilot experiments indicating a theta peak of the prefrontal signal as extracted with our spatial filters around 6–7 Hz, which was confirmed in the final results (see section “Results,” Figure 2). A similar procedure was used previously for real-time estimation of sensorimotor μ-oscillation phase in the alpha band (Zrenner et al., 2018), but further adapted as described below.

Additional constraints were implemented to take into account the presence of muscle and eye blink artifacts when targeting more frontal sources and the intrinsic fluctuations characteristic to the theta oscillation. The algorithm included the following constraints to prevent inappropriate triggering of TMS: (1) Eye movement detection: The low frequency and high amplitude of eye movements and blinks could bias the phase detection algorithm and lead to inappropriate triggering. Eye blinks were detected by determining the maximum range within a 50 ms sliding window of the voltage potentials between four sensor pairs around the eyes (EOG1-Fp1, EOG1-Fp2, EOG2-Fp1, EOG2-Fp2), and taking the sum, with a threshold criterion of 250 μV. Therefore, TMS triggers were blocked for the following 700 ms after an eye blink was identified. (2) Muscle artifacts: If any of the EEG channels exceeded a range threshold within the window of analysis, signal quality was deemed to be affected by cranial muscle or movements artifacts. General EEG artifacts were detected when any channel exceeded a maximum range within a sliding window of 100 ms. This threshold was adjusted manually during the measurement due to fluctuations in the signal’s amplitude during the experiment. (3) Phase stability of theta oscillation: constraints were added for the system to only send a trigger if no phase reset was detected in the previous 500 ms. Phase reset was determined by analyzing phase progression in a sliding 1 s window as follows: The signal was downsampled to 250 Hz, band-pass filtered in the theta range (5–8 Hz, FIR filter order 80, forward and backward), and converted to an analytic signal using the Hilbert transform. Instantaneous phase was unwrapped and (accounting for edge effects from the band-pass filter) instantaneous frequency was determined from phase progression over discrete 16 ms steps (units of Hz). The average squared difference between subsequent instantaneous frequencies across 16 ms steps was calculated and used as an “oscillation stability” criterion for the real-time phase-detection. (4) Theta amplitude: A user adjustable amplitude threshold prevented application of stimuli during periods where no reliably detectable theta oscillation was present. During the experiment, the general EEG artifacts and amplitude threshold were adjusted manually to track fluctuations and maintain a consistent stimulation rate. The implementation of the manually adjusted threshold was necessary as the intensity of the EEG background noise shifted throughout the experiment, probably due to change in the impedance of the electrodes and subjects’ muscular activity, which changes the profile of spectral power and limits the possibility of establishing a static threshold. For the real-time phase-detection, we set the amplitude threshold to the mean of the minimum and maximum power of the theta band. For the *post hoc* comparison of different algorithms, the theta amplitude threshold was set as the 50% quantile with regards to the whole signal.

### Simulating the Performance of the Phase Estimation Methods

In order to investigate the accuracy of the phase estimation method with different sets of parameters, we used the resting-state EEG data and applied the algorithm described above post-hoc, mimicking the real-time situation. This was performed by overlapping segments of 1,024 ms duration which were selected every 5 ms, and having the phase corresponding to the last sample of each segment estimated using the same procedure as described in the preceding section. The resulting estimated phase was then compared with the “gold-standard phase,” obtained by using the whole signal, which involved data before and after each time point of interest (zero-phase forward and backward 5–8 Hz FIR band-pass filter of order 1,000, and Hilbert transform). The difference between the estimated and the gold-standard phases serves as an error measure to compare the accuracy of different real-time phase estimation methods. We also calculated the proportion of instances when the distance between the estimated phase and the gold-standard phase was less than 45° to further quantify the accuracy of the phase estimation methods. Also, the “gold standard phases” were compared between spatial filters, calculating the correlation coefficient between the phases of the signal yielded by each spatial filter at individual level, as an estimate of phase agreement of the signal from these filters. The resulting correlation coefficients were then statistically compared.

Phase estimation error from the real-time phase estimation was assessed *post hoc* for signals extracted using the spatial filters. Then, for the signal extracted using the individual filter W_ind_, the impact of additional constraints on phase estimation accuracy was assessed comparing the following conditions: individual source based spatial filter without constraints W_ind_, as above; application of the phase stability constraint; application of the theta amplitude constraint; application of both the phase stability and amplitude constraints.

### Real-Time Closed-Loop Data Processing Set-Up

Real-time data acquisition, data processing and TMS trigger control were implemented using a custom-built dedicated digital biosignal, executed on a dedicated xPC Target PC running the Simulink Real-Time operating system (DFI-ACP CL630-CRM mainboard). For the purpose of the real-time phase detection, EEG data was sent to a real-time processor through a real-time UDP interface at a packet rate of 5,000 Hz (one sample per channel) (Zrenner et al., 2018). The signal from left DMPFC was extracted using individual spatial filters based on LCMV beamforming (filter W_ind_), for which all electrodes were used, with the exception of the ones in the outer rim (electrodes with labels 9 and 10 in the International 10–5 EEG system).

### Statistics

All statistical analyses were performed using MATLAB R2018b (Mathworks Ltd., United States), first involving the assessment of the data distribution’s pattern. Data following a normal distribution were analyzed using parametric methods (ANOVA, followed by *post hoc* pairwise comparisons when appropriate). Data not following a normal distribution were log-transformed and analyzed using parametric methods, as above, in the case the log-transformed distribution was normal. When this was not the case, the original data was then analyzed using non-parametric methods (Kruskal-Wallis test, followed by *post hoc* pairwise comparisons when appropriate). The correlation analyses were performed using Pearson correlation. Phase accuracy is reported as circular standard deviation. Threshold for statistical significance was set as p < 0.05.

## Results

**FIGURE 1:**
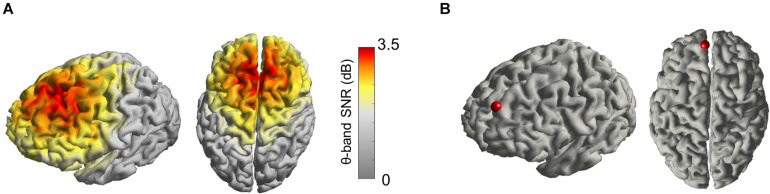
**(A)** Average distribution of the signal-to-noise ratio (SNR) of the theta oscillation projected in the source space, including all 18 subjects, plotted on an averaged cortical model. **(B)** Cortical site (red dot) set as the region of interest for the EEG source activity estimation, centered on the left DMPFC.

From the 8-min resting-state EEG signal, we observed that the spectral power in the theta band was more prominent in prefrontal regions, as expected (Figure 1A). Given the particular interest in the DMPFC for its role in cognition and as possible anatomical target for brain stimulation interventions, we designed it as the source of the signal of interest for the calculation of the filter W_ind_ (Figure 1B).

**FIGURE 2:**
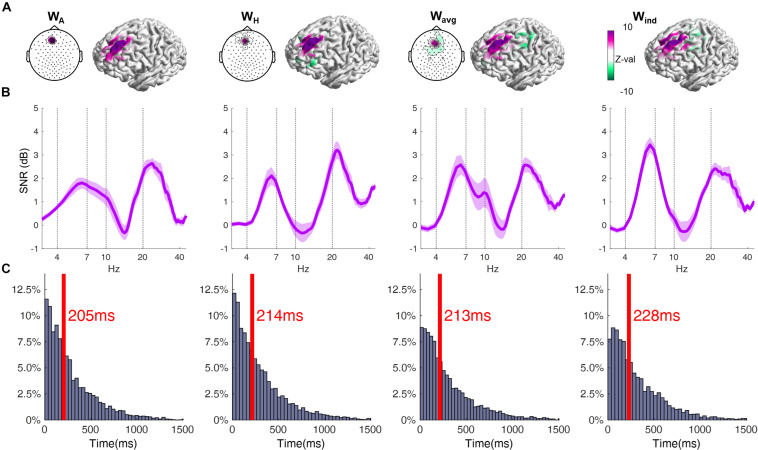
**(A)** Topographical plot displaying the EEG channels’ coefficient weighs of the respective filter. Cortical surface plots show the sensitivity profile of the respective filter, averaged across all subjects. The coefficient weights are given in arbitrary units, and are here normalized across all individuals using the standard score (z-value). Note that for the W_ind_ filter the values are different for each subject, and its average is depicted in W_avg_. Also, results using the W_ind_ filter involved the application of the individual filter for each subject, and thus cannot be shown as a single topographical plot. **(B)** Power spectra of the resting-state EEG signal, obtained by using the respective spatial filters, averaged across all subjects (shaded area corresponds to ± 1 SEM). Data is depicted in form of Signal-to-Noise-Ratio. (ANOVA, *p* = 0.0055; *post-hoc* W_A_ = W_H_ < W_ind_). **(C)** Distribution of the time lengths of epochs between phase slip events of the theta oscillation. Red line and text indicate the median of the respective distribution (ANOVA, *p* = 0.0009; *post-hoc* W_A_ < W_H_ = W_avg_ < W_ind_).

### Performance of Spatial Filters

The average of the W_ind_ filters across all subjects had high amplitude positive coefficients in a central group of electrodes centered around AFFh1, Fz and F1, vs. a surrounding area of electrodes with negative coefficients (Figure 2). Accordingly, the average of the sensitivity profiles shows that the W_ind_ filters are particularly sensitive to the anterior part of the left superior gyrus, corresponding to the left DMPFC, as expected. Slight inter-individual differences in the W_ind_ filter’s coefficient weights conformation and the respective cortical sensitivity profile can be seen in the individual data (see individual filters in Supplementary Material). When comparing different spatial filters, at first glance, the sensitivity profiles of all filters appear to share the same characteristics, with higher sensitivity to the region around the left DMPFC. However, by basing this on a grand-average result, we might miss relevant differences in the individual results. To account for that, we performed an intra-subject correlation analysis of the sensitivity profiles and compared the resulting correlation coefficients. This revealed a significantly lower correlation between the sensitivity profiles from the W_ind_ filter and other filters, compared to the correlation between these other filters (Table 1). This suggests that, on an individual scale, the individual filters W_ind_ are more sensitive to different regions of the cortex, compared to the non-individual filters.

**TABLE 1:** Correlation matrix showing the comparison of the correlation coefficients between sensitivity profiles from the respective filters, averaged across all subjects (ANOVA, p = 0.0025; post-hoc [W_ind_ vs. W_A_, W_H_, W_avg_] < [W_H_ vs. W_A_]† < [W_avg_ vs. W_A_, W_H_]††).

	W_A_	W_H_	W_avg_	W_ind_
W_A_	1	0.69^†^	0.73^†⁣†^	0.48
W_H_		1	0.74^†⁣†^	0.46
W_avg_			1	0.55
W_ind_				1

This particularity of the W_ind_ filter is probably responsible for considerable differences in the spatially filtered signal, compared to other filters, as observed in the power spectra of the yielded signal. Using only a single electrode as source (W_A_), the resulting average power spectrum reveals the theta oscillation with little distinction with the alpha oscillation, both with lower power than higher frequency beta oscillations (Figure 2A). Using a Hjorth montage centered around that channel (W_H_) successfully suppresses the alpha, but with little gain to the SNR of theta (Figure 2B). Using the average of the individual filters, there is some gain in the theta SNR (W_avg,_ Figure 2C). It is only when using the respective individual filters W_ind_ that we obtain a significantly higher SNR in the theta band (Figure 2D). On an important note, here we observe that, regardless of the spatial filter used, the average frequency peak of the local theta oscillation is around 6–7 Hz. The phase correlation was highest between the W_avg_ and W_ind_ filters, followed by W_H_ and other filters, and the lowest phase correlation being between W_A_ and other filters (Table 2).

**TABLE 2:** Correlation matrix showing the correlation coefficients of the phases of prefrontal theta oscillation in the signal resulting from the respective filters, averaged across all subjects (Kruskal-Wallis test, p < 0.0001; post-hoc [W_A_ vs. W_H_, W_avg,_ W_ind_] < [W_H_ vs. W_avg_, W_ind_] † < [W_avg_ vs. W_ind_] ††).

	W_A_	W_H_	W_avg_	W_ind_
W_A_	1	0.02	0.05	0.03
W_H_		1	0.15^†^	0.12^†^
W_avg_			1	0.25^†⁣†^
W_ind_				1

The analysis of the stability of theta oscillation also shows difference between the yielded signal from the spatial filters. The intervals of theta oscillation between phase-slips were significantly longer in the signal from the individual source-based spatial filter. Interference from other cortical oscillations could have affected the phase progression estimation of the theta oscillation, leading to an overestimation of phase slips and thus shorter epochs between these events, especially in the signal from non-individualized filters (Figure 2).

### Spatial Filters and Theta Oscillation Constraints in the Phase-Detection Algorithm

Here we investigate the procedures that may optimize the accuracy of the phase-detection method. We define the “accuracy” as deviation of the phase indicated by the real-time algorithm with respect to the “gold-standard phase,” with higher accuracy representing higher overall agreement between these phases.

**FIGURE 3:**
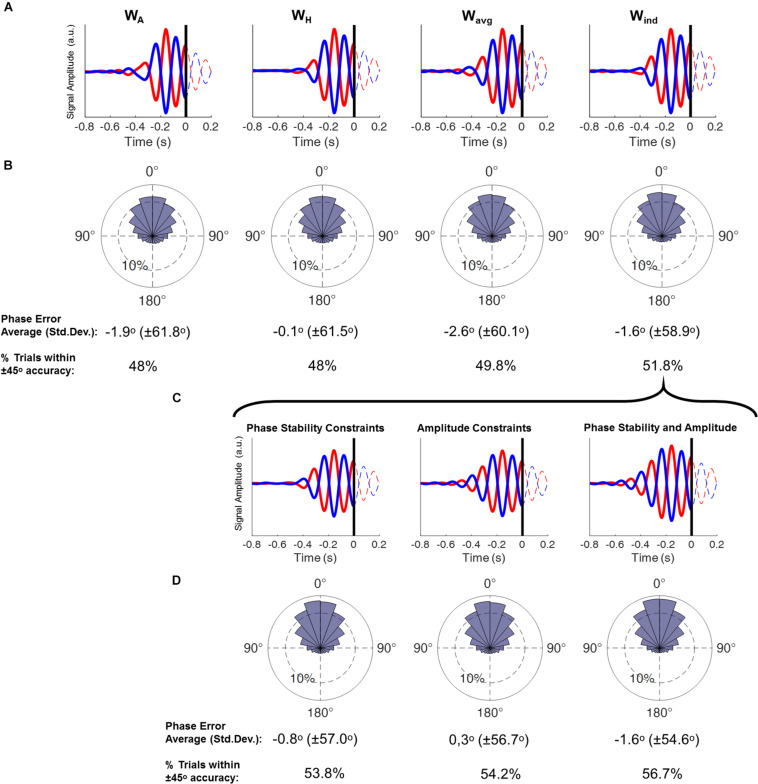
**(A)** Time course plots display the averaged epochs obtained by running the real-time phase detection algorithm with resting-state EEG, with data obtained by using each spatial filter. Here only trials that were classified as positive peak (red) and negative peak (blue) were included. Time = 0 refers to the time point the theta phase was being estimated. Columns display the results from each spatial filter. **(B)** Phase histograms show the accuracy of the phase estimation from each spatial filter, obtained by subtracting the estimated phase of all epochs by their corresponding “gold-standard” phase (phase-bin width 20°, inner ring corresponds to 10% of the total trials). Below are the circular averages and standard deviations of the results, and the proportion of epochs within ± 45° accuracy (ANOVA, *p* = 0.0025; *post-hoc* W_A_ = W_H_ < W_avg_ < W_ind_). **(C)** Time course plots display the averaged epochs obtained by running the real-time phase detection algorithm with resting-state EEG (as in **A**), with data obtained by using the W_ind_ filter and followed by different signal constraints: Phase stability, signal amplitude and both phase stability and amplitude constraints. **(D)** Phase histograms show the accuracy of the phase estimation (as in **B**) from the signal after application of each constraint (ANOVA, *p* = 0.0002; *post-hoc* [NO constraints] < [phase stability] = [amplitude] < [phase stability AND amplitude]).

The choice of spatial filter significantly impacted the phase estimation, with increased accuracy observed when using the W_ind_ filters, followed by the use of W_avg_ filters, and with the lowest accuracy when using the W_A_ and W_H_ filters (Figure 3). Note that the difference is not in terms of the average phase error itself (i.e., the average difference between the estimated phase and the gold-standard) but in the standard deviation of that difference. In other words, on average all conditions have a very high accuracy, with the error close to 0 degrees. However, higher standard deviations mean that a larger proportion of trials had a higher phase estimation error. This can be exemplified by calculating the proportion of instances where the phase error exceeded the pre-established threshold. Setting a limit of ± 45°, we observe that either when using the W_A_ and W_H_ filters, 48% of the phase estimations are within that limit, whereas when using the W_ind_ filter, this number rises to 52% (Figure 3).

A limitation of this method is that the “gold-standard” signal was different for each filter. Therefore, each of the phase-detection simulations was compared with respect to the “gold-standard” signal yielded by the same filter. As a consequence, the method does not take into account the possibility that the yielded signal, instead of detecting the actual prefrontal theta, might correspond to other oscillatory modes from different cortical regions. Therefore, if we were to assume that the W_ind_ is more sensitive to the true underlying prefrontal theta, the actual increase in the phase accuracy by using the W_ind_ in comparison to filters W_A_ and W_H_ might be much larger. This possibility is strengthened by the findings of low correlation between the filters’ sensitivity profiles, and also by the lower phase stability of the theta oscillations yielded by non-individualized filters (Figure 2). Finally, we investigated the correlation between the phases of the theta oscillation at the same given epochs in the signal from these filters, and observed a low correlation between the theta phases from the W_A_ and the W_H_ compared with other filters, with the highest agreement being between W_avg_ and W_ind_, further suggesting that other oscillations of non-interest are confounding the phase estimation when non-individual filters are used to extract the signal.

We also aimed to further increase the accuracy of the phase-detection by taking into account the dynamics of the prefrontal theta oscillation, which involved adding the phase stability and amplitude threshold constrains to guarantee that the phase-detection would be performed during stable signal segments. These constrains were observed to individually contribute to the increase of the phase-detection accuracy (Figure 3).

### Performance in Real-Time

**FIGURE 4:**
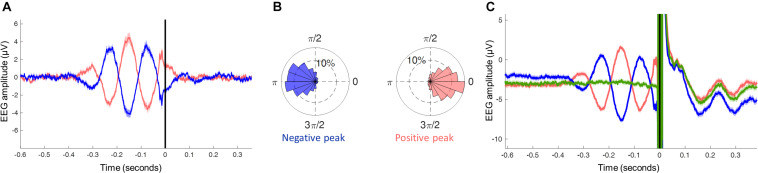
**(A)** EEG signal generated by the individual filter W_ind_, averaged across all subjects and trials around the trigger (time 0 s) located by the phase detection algorithm in the non-stimulated trials (red: positive peak; blue: negative peak). **(B)** Phase histogram of the estimated phases of the oscillation in the theta band at the time of the trigger, divided by trials triggered on the negative peak and positive peak (phase-bin width 20°, inner ring corresponds to 10% of the total trials). **(C)** EEG signal generated by the individual filter W_ind_, averaged across all subjects and trials around the trigger (time 0 s) located by the phase detection algorithm in the stimulated trials (red: positive peak; blue: negative peak; green: random phase). Note the large TMS artifact and TMS-evoked EEG potentials.

As a proof of concept, we proceeded to applying the real-time phase-detection algorithm using the specifications above, including the W_ind_ filter as well as phase stability and amplitude constraints, in order to deliver theta phase-specific TMS pulses to the left DMPFC. By analyzing the resulting signal from the real-time phase-detection, we observed that the averaged signal of the pre-stimulus epochs closely resembles the simulations shown in Figure 3, with at least 2 distinct theta cycles observed prior to the trigger, peak amplitude between −200 to −100 ms with respect to the TMS trigger (Figure 4A). By estimating the theta phase using whole epochs, “gold-standard phase,” we can observe the phase of the theta oscillation where each trigger was placed, thus allowing proper estimation of the real-time phase-detection accuracy. The results showed values similar to the accuracies obtained in the simulation, with 55.7% of the estimations being within ± 45° accuracy in the negative peak, and 56.4% in the positive peak (Figure 4B).

When observing the signal in the epochs where real TMS was applied, we notice that the averaged pre-stimulus signal resembles the simulations and the non-stimulated epochs, as expected. Moreover, we also see a massive electrical artifact caused by the TMS pulse in the EEG and the consequent brain response to direct stimulation. These can severely distort the phase estimation, which is the reason behind the need for accuracy tests to be performed on non-stimulated epochs, marking the EEG as if a real TMS pulse was applied.

## Discussion

The objective of this study was to develop a method to apply TMS phase-locked to the ongoing theta oscillation of the left DMPFC of healthy human subjects. We have adapted our previous algorithm, designed to detect the phases of the sensorimotor μ-rhythm in real-time, to now trigger stimuli phase-locked to the ongoing prefrontal theta oscillation. Importantly, this was possible by taking into consideration the individual anatomical location of the signal’s source and the particularities of the theta oscillation dynamics.

For this purpose, we used individual filters based on source for extracting the EEG signal in real time from the region of interest. The higher SNR in the theta band of the signal yielded by the individual filters suggest a higher accuracy in detecting the signal from the DMPFC (Figure 2). Moreover, the estimated sensitivity profile of the individual filters presented a low correlation with other filters. This suggests that individual differences in the variables used to produce the filter (cortical anatomy, EEG electrodes position over the scalp, resting-state EEG signal and its covariance matrix) are relevant to be taken into account in designing a spatial filter to detect prefrontal theta oscillation. This personalized approach might have been responsible for a significantly higher accuracy in the real-time phase detection algorithm when this filter was applied (Figure 3), as the higher SNR in the theta band provided by this filter is expected to increase the accuracy of phase detection compared to other filters (Zrenner et al., 2020). However, for the purposes of estimating the phase accuracy, the signal for both the phase-detection simulation and for obtaining the “gold-standard phases” were produced by the same respective filter being examined. This means that the estimated accuracy does not account for the possibility that other oscillatory activities might be overriding the phase-detection, meaning that the phase inaccuracy with respect to the “real prefrontal theta” might be even greater. Properly identifying the “real theta” originating from the DMPFC would need to involve invasive electrophysiological recordings, and is beyond our present possibilities. Nevertheless, we can estimate a possible best candidate based on the indirect evidences available. At first, assuming that the preferential oscillatory mode region of interest is in the theta band, we can suppose that both the individual filters and its grand average are likely to be more accurate in extracting the “real prefrontal theta” than the other filter options, given the higher SNR of that oscillation in the yielded signal. This is reflected in the higher correlation between the phases of the theta oscillation of the signal yielded by these filters, with little agreement with the phases produced by the Hjorth montage and even smaller when using a single electrode, further suggesting that the signals being enhanced by these filters are of different origin. Finally, the lower stability of theta oscillations observed in the signal from non-individual filters suggests the existence of considerable interference with other oscillatory activities, falsely resembling theta phase slips. Conversely, longer durations of stable theta oscillation epochs were observed using the individual filter, which are closer to what has been reported in a previous study using invasive cortical recordings, with theta oscillation durations of on average 650 ms (Kahana et al., 1999). These reported values, however, are far above what we obtained, which is expected given that the signal was obtained during the execution of a continuous visuospatial task, which is more likely to recruit more stable theta oscillation, compared to the resting state used in our experiment. Moreover, the values reported in the aforementioned study were obtained through invasive recordings, which provides considerable protection from contamination from distant oscillatory signals compared to scalp EEG. Interestingly, the upper limit (95% percentile) of stable theta epochs was found to be around 1,500 ms in both our results and the aforementioned report (Kahana et al., 1999).

Taking into consideration the transient nature of the theta oscillation also significantly increased the method’s accuracy. Creating constraints to avoid triggering during epochs of low theta power or theta phase shifts were independently responsible for increasing the accuracy (Figure 3). The relevance of these phenomena can also be seen in the final results: When averaging the non-stimulated trials, at least two cycles of an oscillation in the theta frequency-band prior to the stimulus marker can clearly be identified (Figure 4C). It should be noted that phase-locking to the sensorimotor μ-rhythm yields a continuous oscillation pattern, which extends up to 4–5 cycles prior to the trigger (Zrenner et al., 2018). This is not expected in phase-locking to the prefrontal theta rhythm, as this oscillation, as already mentioned, was found to occur in well-defined epochs of only a few hundred milliseconds, prone to sporadic shifts in amplitude and phase resetting (Kahana et al., 1999; Rizzuto et al., 2006; Rutishauser et al., 2010), resulting in the oscillatory activity averaging out the further it is from the time point of interest (TMS trigger). The application of these constraints was necessary to properly achieve accurate real-time phase-detection of the prefrontal theta oscillation. The resulting algorithm was found to be effective, with its accuracy in triggering at the desired phase being comparable to previously published phase-triggering algorithms (Siegle and Wilson, 2014; Blackwood et al., 2018; Zrenner et al., 2018; Madsen et al., 2019), and in line with the limitations imposed by the SNR of the data (Zrenner et al., 2020).

A limitation of this study is that we did not analyze the response signal produced by the different stimulation conditions. Although the stimuli applied to different phases of theta might have led to different cortical responses, it is exceedingly challenging to investigate these differences in the EEG signal response, given that the ongoing oscillations influence the resulting signal (Desideri et al., 2019). Differences in stimulating opposite phases of the prefrontal theta oscillation could be better detected by methods unbiased by the pre-stimulus EEG, such as functional MRI or near-infrared spectroscopy. Another unbiased output is behavioral performance. It has been shown that TMS pulses applied during different phases of the prefrontal theta oscillation have different effects on cognition. More precisely, after applying a series of TMS pulses to subjects performing a working memory task, the accuracy of trials was influenced by the phase of the prefrontal theta during which the TMS pulse was delivered (Berger et al., 2019). Although that study relied on estimating the phase of each trial *post hoc*, by using the method described here it is possible to investigate the effects of cortical stimulation during specific theta phases on cognition in real-time. The method can also be applied in differentially modulating cortical plasticity. The particular role of the theta rhythm in frontal cortex neuroplasticity has inspired the development of a stimulation protocol that delivers repetitive TMS bursts of 50 Hz at a carrier frequency of 5 Hz, and was termed accordingly theta-burst stimulation (TBS) (Huang et al., 2005). Nevertheless, despite the clinical success of TBS, it has not been shown to be superior to standard repetitive TMS protocols (Blumberger et al., 2018). One possible reason is that, although the stimulation is applied in a theta-frequency band, it does not take into account the phase of the ongoing endogenous oscillation. Future studies should determine whether EEG-informed brain-state-dependent repetitive TMS, targeting, e.g., the negative peak of the theta-rhythm in prefrontal cortex indeed leads to neuroplastic changes that are significantly different when compared to random-phase stimulation. The capability of applying repetitive theta phase-locked cortical stimuli demonstrated in this study could potentially be used as neuroplasticity inducing non-invasive brain stimulation, with potential clinical applications.

## Conclusion

Results support the feasibility of synchronizing TMS accurately to a specific phase of the local theta oscillation in DMPFC informed by EEG data analyzed in real time and source space. They may also be relevant for devising EEG-informed personalized therapeutic repetitive TMS protocols for effective treatment of neuropsychiatric disorders.

## Data Availability Statement

The raw data supporting the conclusions of this article will be made available by the authors, without undue reservation.

## Ethics Statement

The studies involving human participants were reviewed and approved by Ethik-Kommission an der Medizinischen Fakultät Eberhard-Karls-Universität Tübingen. The patients/participants provided their written informed consent to participate in this study.

## Author Contributions

CZ, PG, and UZ conceived the study and designed the study protocol. CZ, PG, and BZ set-up the experiment and obtained ethical approval. PB, CZ, MS, and PG designed the algorithms for experiments and analyses. PB created the headmodels. PG and SD conducted the experiments and analyzed the data. CZ, PG, and BZ performed the spectral analysis. All authors contributed to the writing of the manuscript.

## Conflict of Interest

PG, CZ, and BZ report funding through the EXIST translational research program from the German Federal Ministry for Economic Affairs and Energy, with the goal of commercializing the real-time EEG-TMS device used in this study and CZ and BZ report an interest in and employment by the spin-off company resulting from this grant (sync2brain GmbH, Tübingen). UZ received grants from the German Ministry of Education and Research (BMBF), European Research Council (ERC), German Research Foundation (DFG), Janssen Pharmaceuticals NV and Takeda Pharmaceutical Company Ltd., and consulting fees from Bayer Vital GmbH, Pfizer GmbH, and CorTec GmbH, all not related to this work. The remaining authors declare that the research was conducted in the absence of any commercial or financial relationships that could be construed as a potential conflict of interest.
