# Supplementary material for: Prefrontal Theta-Phase Synchronized Brain Stimulation With Real-Time EEG-Triggered TMS
Source: Front Hum Neurosci. 2021 Jun 21;15:691821. doi: 10.3389/fnhum.2021.691821 (PMC8255809; doi:10.3389/fnhum.2021.691821)
Supplement: Supplementary file 1 [file Data_Sheet_1.PDF]

Supplementary Results: Individual spatial filters.

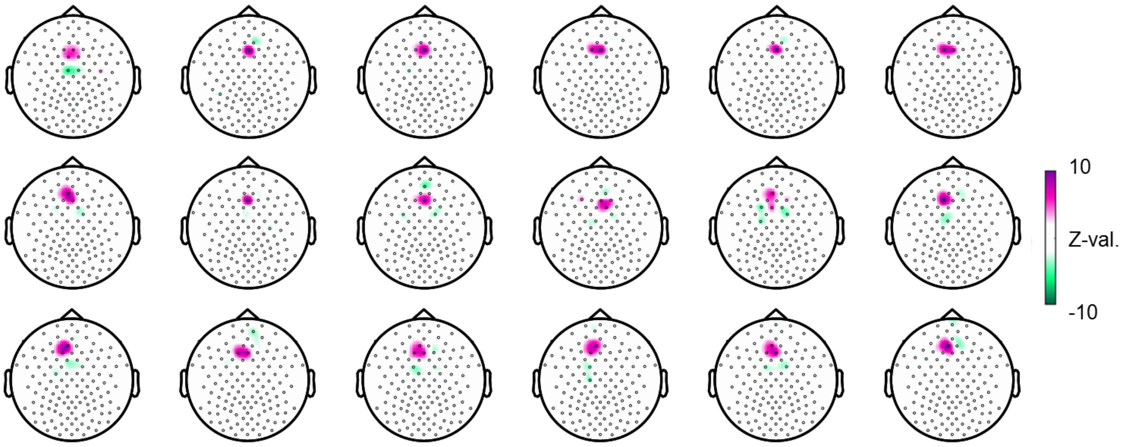

Figure S1: Topographical plots displaying the EEG channels' coefficient weighs of the individual source based filter, each corresponding to a single subject. The coefficient weights are given in arbitrary units, and are here normalized across all individuals using the standard score (z-value).

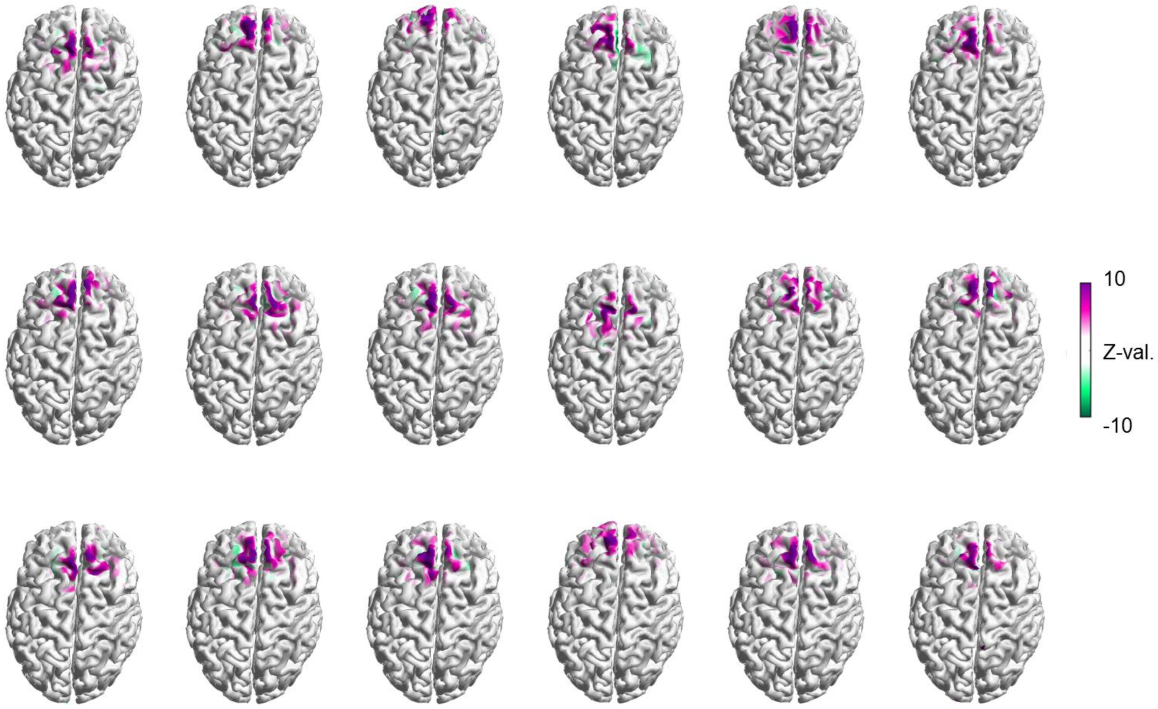

Figure S2: Cortical plot displaying the sensitivity profile of the $\mathbf{W_A}$ filter (only AFF1h channel) at individual level, plotted on an averaged cortical model.

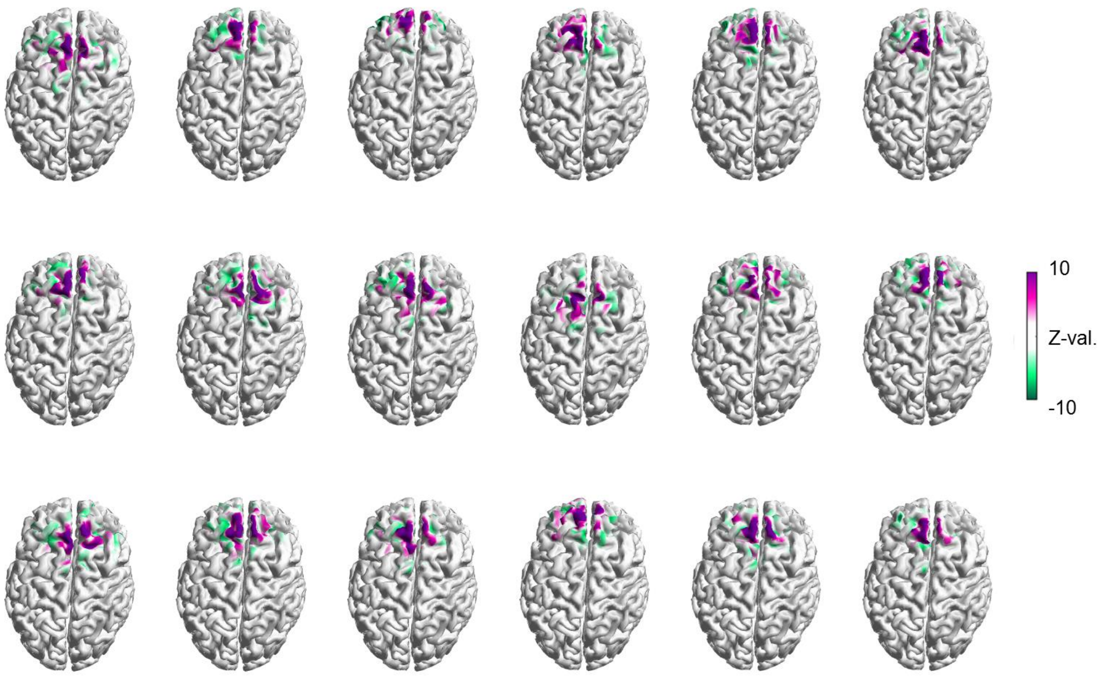

Figure S3: Cortical plot displaying the sensitivity profile of the $\mathbf{W_H}$ filter (Hjorth montage centered around AFF1h channel) at individual level, plotted on an averaged cortical model.

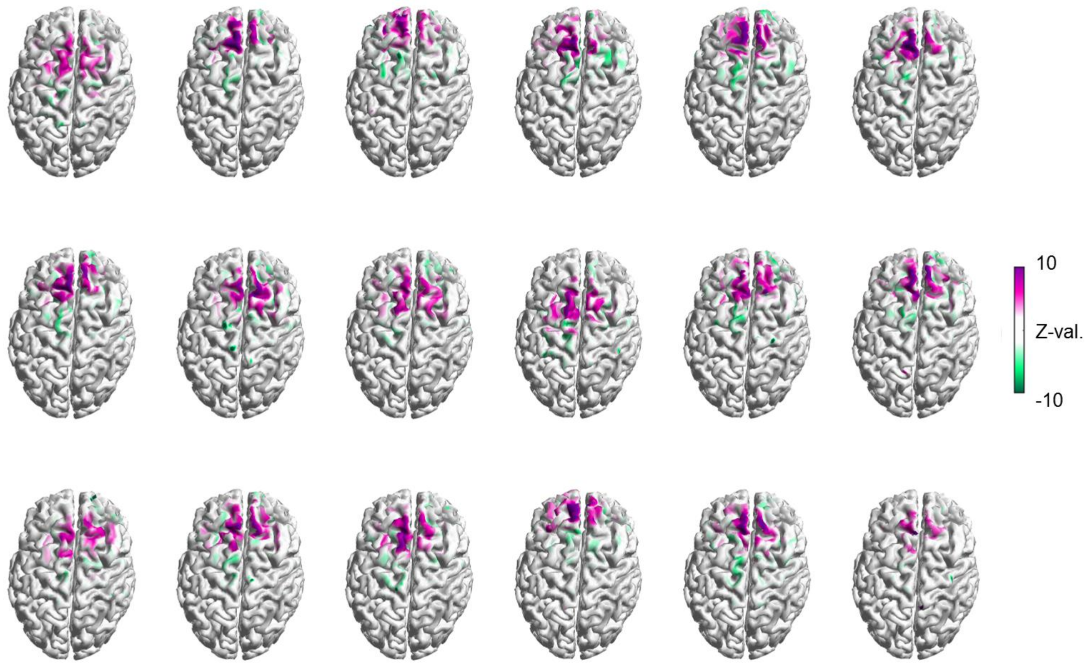

Figure S4: Cortical plot displaying the sensitivity profile of the $\mathbf{W_{avg}}$ filter (grand average of the individual source-based filters across all subjects, applied as a single filter to all subjects) at individual level, plotted on an averaged cortical model.

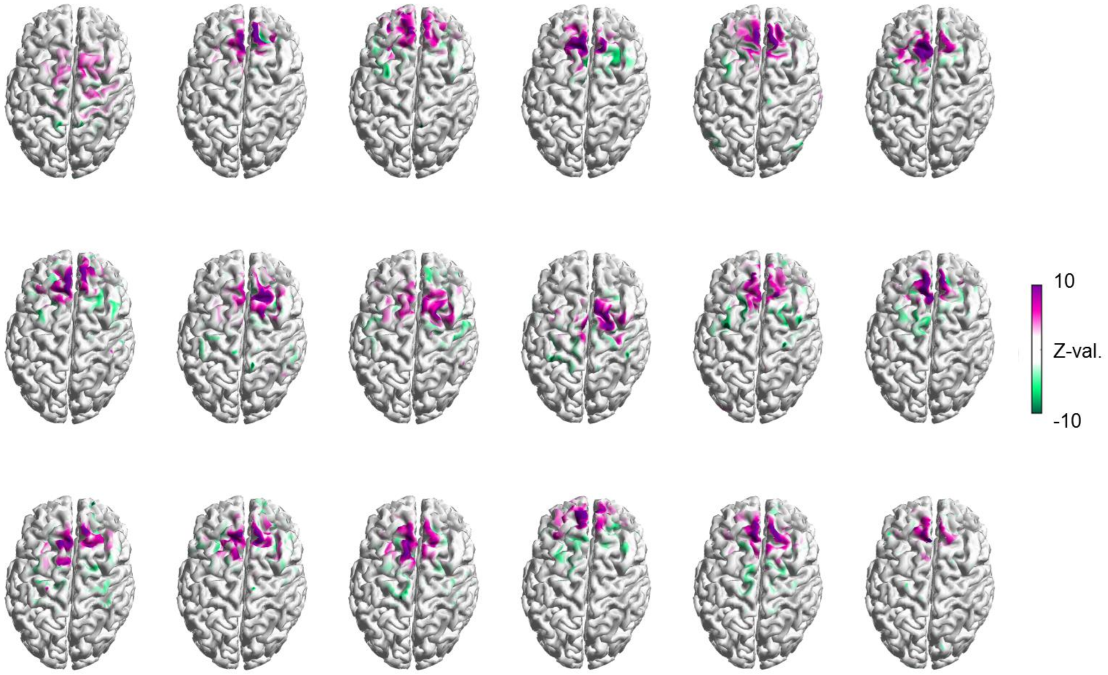

Figure S5: Cortical plot displaying the sensitivity profile of the $\mathbf{W}_{\mathbf{ind}}$ filter (individual source-based filters) at individual level, plotted on an averaged cortical model.

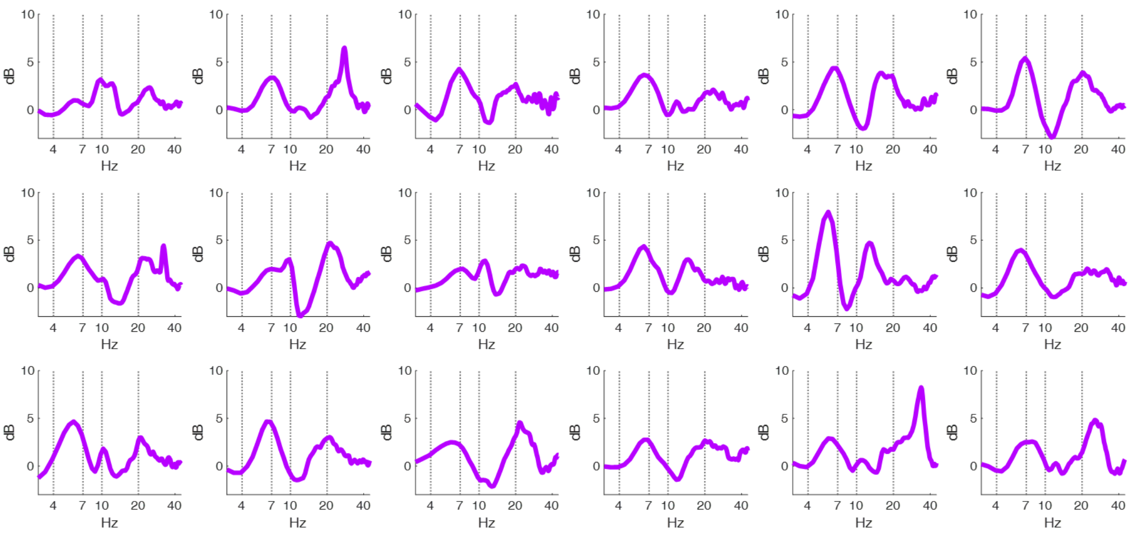

Figure S6: Power spectra of the resting-state EEG signal, obtained by using $\mathbf{W}_{\mathbf{ind}}$ (individual source-based filters) presented in form of Signal-to-Noise-Ratio, each display corresponding to a single subject.
